# The Role of Abscisic Acid in the Regulation of Plasmodesmata and Symplastic Intercellular Transport

**DOI:** 10.1093/pcp/pcz046

**Published:** 2019-03-07

**Authors:** Yoselin Benitez-Alfonso

**Affiliations:** Centre for Plant Science, School of Biology, University of Leeds, Leeds, UK

Coordination of local and systemic responses to environmental cues is mediated by the intercellular transport of small and large molecular signals. In plants, intercellular channels named plasmodesmata (PD) are formed, consisting of a plasma membrane and an appressed ER (desmotubule) traversing cell walls (Cheval and Faulkner 2018; Otero et�al. 2016). These channels provide symplastic (cytoplasm-to-cytoplasm) connectivity between neighbouring cells through which transcription factors, RNAs and other regulatory molecules move to function in a non-cell autonomous manner. Cell walls around PD tightly regulate their transport capacity by constricting the channel aperture and/or width. This regulatory mechanism is mediated by callose, a β-1, 3 glucan polysaccharide that accumulates in cell walls around the PD. PD-associated defects in callose synthesis (mediated by callose synthases, CALS) and/or degradation (mediated by family 17 glycosyl hydrolases also named β-1, 3 glucanases, BGs) alter symplastic transport leading to defects in cell fate specification, organ growth and developmental transitions (Otero et�al. 2016). Mutants for callose synthesis/degradation are also impaired in response to abiotic stresses and during the establishment of pathogenic and mutualistic symbiotic interactions (Cheval and Faulkner 2018; Gaudioso-Pedraza et�al. 2018). Variations in PD number and architecture occur in different cell types and developmental stages, and this is linked to changes in symplastic intercellular transport, although the underlying molecular mechanisms are virtually unknown.

Recent research implicates phytohormones and certain metabolites in the control of symplastic transport (Han and Kim 2016). The role of abscisic acid (ABA) in regulating callose synthesis/degradation and symplastic transport, in the context of intercellular virus spreading and bud dormancy, has been recently reported and reviewed (Alazem and Lin 2017; Tylewicz et�al. 2018). ABA levels are important in coordinating plant defence and developmental responses to environmental cues, thus a mechanistic understanding of the pathways that they activate is essential.

In this issue, Kitagawa et al. dissect the effect of ABA on PD structure and symplastic connectivity in protonema cells of the moss *Physcomitrella patens* (Kitagawa et�al. 2019). The authors use photoactive Dendra2 as a symplastic reporter combined with structural analysis of PD using transmission electron microscopy (TEM) and aniline blue staining to determine changes in callose accumulation. In response to ABA, callose accumulation was detected in the cross-walls of protonema cells for a longer timeframe (∼120h) than the observed restriction in the transport of Dendra2 (∼1-3h). These short-term effects of ABA on symplastic communication were reversible and correlated with a significant reduction in PD width and/or diameter whereas callose levels remained unchanged. In *ppabi1*, a mutant with depleted ABA levels, Dendra2 moves faster and PD densities were slightly increased. Based on these results, the authors concluded that high ABA levels cause a restriction in the symplastic communication between moss protonema cells by a mechanism that induces short-term modifications in PD structure independent of callose accumulation (Kitagawa et�al. 2019).

Previous research described the effect of ABA on virus resistance in plants and this was tightly correlated with changes in the expression of genes involved in callose synthesis/degradation and in symplastic communication (Alazem and Lin 2017). Specifically, it was established that inducing ABA-signaling causes a down-regulation of BGs (the enzymes that degrade callose) leading to restricted viral cell-to-cell movement. A similar mechanism was proposed to mediate the defence response to the fungal pathogen *Leptosphaeria maculans* in *Arabidopsis* (Oide et�al. 2013). In hybrid aspen, ABA prevents early dormancy release in response to chilling and a short photoperiod by restricting the transport of growth factors (including orthologues of Flowering locus T, FT) into dormant buds via a mechanism that involves the constriction of PD by electron-dense sphincters likely made of callose (Tylewicz et�al. 2018). The ABA-insensitive mutant *abi1-1*, which is compromised in the photoperiodic control of bud dormancy, does not form PD sphincters in response to short days and is de-regulated in a large number of PD transcripts including those involved in callose synthesis and degradation at the PD. Moreover, ectopic expression of PDLP1 (a PD-located receptor like kinase that induces callose deposition) was sufficient to restore PD closure and bud dormancy defects in *abi1-1* plants (Tylewicz et�al. 2018).

Downstream of ABA signaling, two components have been characterized to regulate PD response by comparing gene expression profiles in wildtype and *abi1-1* aspen buds (Singh et�al. 2019). These are *PICKLE* (*PKL*, an antagonist of polycomb repression complex 2) and a MADs-box transcription factor orthologous to *Arabidopsis* floral repressor *SHORT VEGETATIVE PHASE* (*SVL)*. In response to high ABA levels (during short days), *PKL* expression is repressed in wildtype but not in *abi1-1*. Downregulation of *PKL* has been shown to be sufficient to complement the formation of PD sphincters and bud dormancy defects in *abi1-1* and restores the expression of *SVL*. SVL binds promoter regions in the sequence of *CALS1* and *GA2 oxidase* genes leading to reduced levels of Gibberellic Acid (GA, a strong growth promoter) and to PD blockage presumably by callose. Both of these ABA-mediated responses are necessary to maintain bud dormancy during short days (Singh et�al. 2019).

Despite strong evidence indicating that PD closure during dormancy is dependent on callose, staining or immunohistochemistry have not been systematically performed. The work by Kitagawa et�al. (2019) indicates that, at least in the short-term, ABA can induce PD constrictions without changes in callose levels and that these constrictions are sufficient to restrict symplastic transport. Coincidently, earlier this year a paper by Wang et al. (Wang et�al. 2019) found that high ABA, GA and auxin levels in endo-dormant buds in the perennial herb *Polygonatum kingianum*, correlate with an increase in the frequency of secondary PDs. Although symplastic communication was not tested, the expression of orthologous genes encoding PD-regulatory factors (including PDLP, the movement protein binding protein 2C, MPB2C and the p-loop containing nucleoside triphosphate hydrolase superfamily protein, EMB) was altered in endo-dormant buds.

Together the data suggest that high ABA levels can have two different effects on PD (Figure�1): induction of PD sphincters by accumulation of callose or changes in PD structure (independent of callose levels) affecting PD number, width and the frequency of secondary PD formation. The occurrence of these responses might depend on the biological context; they might also be temporally and mechanistically related. For example, short term responses to ABA might involve changes in cell wall structure, including callose-cellulose interactions (Abou-Saleh et�al. 2018), leading to immediate changes in PD width, while a more permanent modification in callose synthesis/degradation might be required to maintain PD obstruction for a longer-timeframe. In analyzing the data, crosstalk between hormone-mediated pathways and/or evolutionary differences must be taken into consideration. More detailed time-course analyses of changes in PD structure, cell wall composition and symplastic transport in different ABA synthesis and signalling mutants is required to further elucidate the molecular components underlying these responses. Complementary analysis of mutants of PD proteins regulated in response to ABA can also be informative. Future work in moss will undoubtedly focus on identifying the mobile factors that rely on ABA-mediated regulation of PD to function non-cell autonomously and revealing the evolutionary links underlying this regulation.

**Fig. 1:**
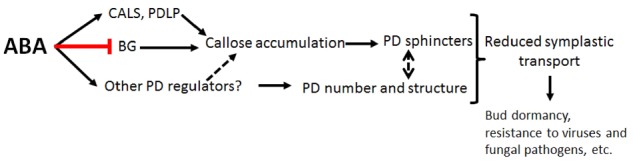
Abscisic Acid-mediated regulation of plasmodesmata and symplastic transport. High Abscisic Acid (ABA) levels regulate the expression of plasmodesmata (PD) proteins involved in callose synthesis/ degradation (such as callose synthases: CALS, PD located protein like kinase: PDLP and β-1, 3 glucanases: BG) among other PD regulators. Callose accumulation at PD leads to the formation of sphincters that restrict symplastic transport. Research from Kitagawa et�al. (2019) and others indicates that changes in PD width, number and frequency of secondary PD (independent of callose levels) also contribute to ABA-mediated responses. How, mechanistically, these changes are orchestrated is not yet known. The outcome, however, is a restriction in the symplastic transport of specific mobile proteins including those that function as growth promoter factors during bud dormancy, or on the cell-to-cell spreading of viruses and other pathogens and/or other environmental responses. Discontinuous arrows show hypothetical relations.
